# Diabetic kidney disease induces transcriptome alterations associated with angiogenesis activity in human mesenchymal stromal cells

**DOI:** 10.1186/s13287-023-03269-9

**Published:** 2023-03-22

**Authors:** Xiaohui Bian, Sabena M. Conley, Alfonso Eirin, Eric A. Zimmerman Zuckerman, Anastasia L. Smith, Cody C. Gowan, Zachary K. Snow, Tambi Jarmi, Houssam Farres, Young M. Erben, Albert G. Hakaim, Matthew A. Dietz, Abba C. Zubair, Saranya P. Wyles, Joy V. Wolfram, Lilach O. Lerman, LaTonya J. Hickson

**Affiliations:** 1 Division of Nephrology and Hypertension, Department of Medicine, Mayo Clinic, 4500 San Pablo Road, Jacksonville, FL 32224 USA; 2 Department of Nephrology, Shengjing Hospital of China Medical University, Shenyang, China; 3 Division of Transplant Nephrology, Department of Transplant Surgery, Mayo Clinic, Jacksonville, FL USA; 4 Division of Nephrology and Hypertension, Department of Medicine, Mayo Clinic, Rochester, MN USA; 5 Department of Laboratory Medicine and Pathology, Mayo Clinic, Rochester, MN USA; 6 Division of Vascular Surgery, Department of Surgery, Mayo Clinic, Jacksonville, FL USA; 7 Department of Orthopedic Surgery, Mayo Clinic, Rochester, MN USA; 8 Department of Laboratory Medicine and Pathology, Mayo Clinic, Jacksonville, FL USA; 9 Center for Regenerative Biotherapeutics, Mayo Clinic, Jacksonville, FL USA; 10 Department of Dermatology, Mayo Clinic, Rochester, MN USA; 11 Department of Biochemistry and Molecular Biology, Mayo Clinic, Jacksonville, FL USA; 12 School of Chemical Engineering/Australian Institute for Bioengineering, The University of Queensland, Brisbane, QLD 4072 Australia

**Keywords:** Chronic kidney disease, Diabetes mellitus, Diabetic nephropathy, Ischemic limb disease, Mesenchymal stromal cells, Regenerative medicine

## Abstract

**Background:**

Therapeutic interventions that optimize angiogenic activities may reduce rates of end-stage kidney disease, critical limb ischemia, and lower extremity amputations in individuals with diabetic kidney disease (DKD). Infusion of autologous mesenchymal stromal cells (MSC) is a promising novel therapy to rejuvenate vascular integrity. However, DKD-related factors, including hyperglycemia and uremia, might alter MSC angiogenic repair capacity in an autologous treatment approach.

**Methods:**

To explore the angiogenic activity of MSC in DKD, the transcriptome of adipose tissue-derived MSC obtained from DKD subjects was compared to age-matched controls without diabetes or kidney impairment. Next-generation RNA sequencing (RNA-seq) was performed on MSC (DKD *n* = 29; Controls *n* = 9) to identify differentially expressed (DE; adjusted *p* < 0.05, |log_2_fold change|> 1) messenger RNA (mRNA) and microRNA (miRNA) involved in angiogenesis (GeneCards). Paracrine-mediated angiogenic repair capacity of MSC conditioned medium (MSCcm) was assessed in vitro using human umbilical vein endothelial cells incubated in high glucose and indoxyl sulfate for a hyperglycemic, uremic state.

**Results:**

RNA-seq analyses revealed 133 DE mRNAs (77 upregulated and 56 down-regulated) and 208 DE miRNAs (119 up- and 89 down-regulated) in DKD-MSC versus Control-MSC. Interestingly, miRNA let-7a-5p, which regulates angiogenesis and participates in DKD pathogenesis, interacted with 5 angiogenesis-associated mRNAs (transgelin/*TAGLN*, thrombospondin 1/*THBS1*, lysyl oxidase-like 4/*LOXL4,* collagen 4A1/*COL4A1* and collagen 8A1/*COL8A1*). DKD-MSCcm incubation with injured endothelial cells improved tube formation capacity, enhanced migration, reduced adhesion molecules E-selectin, vascular cell adhesion molecule 1 and intercellular adhesion molecule 1 mRNA expression in endothelial cells. Moreover, angiogenic repair effects did not differ between treatment groups (DKD-MSCcm vs. Control-MSCcm).

**Conclusions:**

MSC from individuals with DKD show angiogenic transcriptome alterations compared to age-matched controls. However, angiogenic repair potential may be preserved, supporting autologous MSC interventions to treat conditions requiring enhanced angiogenic activities such as DKD, diabetic foot ulcers, and critical limb ischemia.

**Supplementary Information:**

The online version contains supplementary material available at 10.1186/s13287-023-03269-9.

## Background

Tissue hypoxemia from loss of microvascular capillary density is a major contributor to morbidity and mortality in individuals with diabetic kidney disease (DKD), one of the most common causes of kidney failure worldwide [1]. Sustained hyperglycemia and uremia alter vascular function and integrity in the kidney and limbs wherein disease pathogenesis is further fueled by microvascular rarefaction, tissue hypoxemia, sterile inflammation, cellular senescence, endothelial dysfunction, and extracellular matrix deposition [2–6]. These effects culminate in complications common in DKD including end-stage kidney disease (ESKD), cardiovascular disease, critical limb ischemia, and amputations of lower extremities [7]. Despite a 2-decade decline in lower extremity amputations between 2009 and 2015, rates rebounded by 50% in adults with diabetes [8]. Furthermore, the odds of major amputations are nearly twofold higher in individuals with chronic kidney disease (CKD) or ESKD compared to persons without CKD/ESRD [9]. Therefore, therapeutics aimed at optimizing vascular repair and relieving endothelial dysfunction in the DKD patient population are urgently needed.

Regenerative cell-based therapies, such as mesenchymal stem/stromal cells (MSC), have considerable potential to boost endogenous repair of injured tissues. MSC release soluble factors, such as extracellular vesicles (EVs), growth factors, and cytokines, which enhance tissue regeneration and stimulate pro-angiogenic signaling [10–12]. In preclinical models of DKD, renovascular disease, and ischemic limb disease, MSC-based approaches improve kidney function, reduce hypoxemia, and restore microvascular density [2, 11, 13–15]. Collectively, these encouraging studies provided the basis for clinical application of MSC in phase I and II studies in DKD (NCT05362786, NCT02585622, NCT03840343, NCT04869761, NCT04125329, NCT04216849, NCT02008851, NCT03270956, NCT02836574) [16] and diabetic foot ulcers (NCT04464213, NCT04104451, NCT00955669, NCT04466007, NCT02375802, NCT01686139) [17].

Despite the promise of MSC in DKD, numerous challenges remain. Chronic hyperglycemia and uremia may damage endogenous repair systems like MSC, disturbing the coordinated network of pro- and anti-angiogenic factors activated by these cells. Autologous (versus allogeneic) MSC source may be preferable, given reduced risk of allosensitization, particularly for patients that might ultimately require kidney transplantation [18]. However, multiple DKD-related factors including age, hyperglycemia, uremia, cellular senescence abundance, or obesity may alter MSC functionality and number [13, 19–21]. We and others demonstrated that immunomodulatory and paracrine activities of MSC are maintained despite kidney impairment with and without diabetes [21–23]. However, it is unclear whether the microenvironment of DKD negatively influences the angiogenic potential of MSC. Elucidation of pro-angiogenic and tissue-repairing potential of DKD-MSC in the development of MSC-based regenerative medicine strategies for this population is crucial.

Our previous phase 1 clinical trial showed that intra-arterial infusion of autologous adipose-derived MSC (AD-MSC) improved kidney blood flow and reduced kidney tissue hypoxia as assessed through blood oxygen level dependent magnetic resonance imaging (BOLD-MRI) and improved glomerular filtration rate in atherosclerotic renovascular disease subjects [24]. These findings reflected AD-MSC angiogenic capabilities to form new vessels and restore the microcirculation, consistent with our experimental observations in swine atherosclerotic renal artery stenosis [2, 10, 25]. AD-MSC remain a viable cell option for clinical applications given their high abundance in adipose tissue and ease of harvest when compared to bone marrow-derived MSC (BM-MSC). Interestingly, AD-MSC proved more effective than BM-MSC in promoting neovascularization in animal models of ischemic diseases [26, 27]. Given the need for regenerative investigations in CKD, we are currently conducting two phase 1 clinical trials testing AD-MSC (NCT04869761) and BM-MSC (NCT05362786) in the treatment of DKD/CKD. Therefore, based on our prior experiences and the literature, we compared the transcriptome of AD-MSC obtained from DKD subjects and age-matched controls focusing on angiogenesis-related mRNA and miRNA targeting these genes. We further compared the angiogenic repair capacity in vitro of AD-MSC from DKD subjects and controls.

## Methods

### Study participants

The expression of angiogenesis-related mRNAs and miRNAs and angiogenic potential was characterized in AD-MSC isolated from diabetic kidney disease (DKD) subjects. Eligible individuals age ≥ 18 years were recruited from Mayo Clinic in Rochester, Minnesota, in the Nephrology clinic between November 2015–2019 (IRB: 15-000933), as previously reported [21]. Informed consent was obtained by trained clinical research coordinators. DKD was defined as estimated glomerular filtration rate (eGFR) < 60 mL/min/1.73 m^2^ and/or abnormal albuminuria with preserved kidney function (eGFR ≥ 60) in the setting of pharmacologically treated Type 1 or Type 2 diabetes mellitus. Controls were age-matched individuals, without diabetes or CKD, undergoing laparoscopic nephrectomy for kidney donation. Adipose tissue was collected from DKD (*n* = 29) participants in an outpatient surgical suite, while adipose tissue from controls (*n* = 9) was sampled during their surgical procedure. To minimize bias, those with dialysis dependency, kidney transplant, immunosuppressive therapy, hemoglobin A1c (HbA1c) > 11% (97 mmol/mol), or malignancy were excluded. The Mayo Clinic Institutional Review Board approved all experimental study procedures and all participants provided written informed consent prior to participating.

### MSC harvest and characterization

Abdominal subcutaneous adipose tissue (0.5–2.0 g) was collected from control and DKD subjects to isolate MSC as previously described [20, 21, 25]. MSC were cultured in standard conditions (37 °C with 5% CO_2_) in advanced minimum essential medium supplemented with 5% platelet lysate [28] (PLTmax, Mill Creek Life Sciences, Rochester, MN). Primary MSC at passages 3–5 were phenotyped in both groups to confirm cell surface marker expression by imaging flow cytometry (Amnis® FlowSight®, Austin, TX) [19–21]. The following fluorescently labeled antibodies were used: cluster of differentiation (CD)73 (ab106677; Abcam, Waltham, MA), CD90 (ab124527; Abcam), and CD105 (ab53321; Abcam). Conversely, MSC lack expression of CD34 (340441; BD Biosciences, San Jose, CA) or CD14 (ab82012; Abcam). For surface marker expression, 0.25–1.0 × 10^6^ cells were incubated with the antibodies for 30 min at 4 °C according to the manufacturer’s instructions. Following incubation, cell suspensions were washed, pelleted, and resuspended in staining buffer (00-4222-57; Thermo Fisher Scientific, Waltham, MA). Thereafter, samples were run on the FlowSight® and analyzed using IDEAS software version 6.2. Initial gates were selected for single cells (aspect ratio intensity versus brightfield), followed by gates of positively stained cells. Thresholds for positivity were established from scatterplots generated by single-stained compensation beads (A10497, Molecular Probes, Eugene, OR).

### Collection of MSC conditioned medium (MSCcm)

MSC (passages 3–5) were cultured to 70% confluence in T-75 flasks, then washed extensively with phosphate-buffered saline, and replenished with serum-free advanced minimum essential medium for 36 h. After serum starvation, MSCcm was centrifuged at 2000×*g* for 20 min at 4 °C to remove cellular debris, aliquoted and stored at − 80 °C for in vitro studies. MSCcm was normalized by MSC density per sample (1 mL conditioned medium for every 0.13 × 10^6^ MSC) [21]. Thrombospondin 1 (TSP1) protein was measured in MSCcm using a commercially available kit (ELISA; R&D DTSP10) following manufacturer’s protocol.

### MSC mRNA sequencing and data analysis

To explore MSC angiogenic regulation in DKD, high-throughput RNA sequencing was performed as previously reported [21]. Briefly, RNA libraries were generated from 1 µg of total RNAs using an Illumina TruSeq RNA Sample Prep Kit v2 and loaded onto flow cells (8–10 pM) to generate cluster densities of 700,000/mm^2^. MSC were sequenced on an Illumina HiSeq 2000. Comprehensive analysis including alignment, gene and exon quantification, and QC of raw RNA sequencing reads was performed using the Mayo Analysis Pipeline for RNA Seq (MAP-RSeq) version 3.1.3 [29], an in-house bioinformatics workflow. R-bioinformatics package (edgeR) was used for differential gene expression analysis between MSC from DKD and control participants. Genes with *p* adjusted value < 0.05 and |log2FC|> 1 were considered significantly differentially expressed (DE) genes. GeneCards® database (http://www.genecards.org/) was utilized to screen genes associated with angiogenesis. DE angiogenesis mRNAs-of-interest were visualized on volcano plots and heatmaps created using R package ggplot2.

### MSC miRNA sequencing and data analysis

Secondary analyses of high-throughput miRNA analyses on MSC were performed utilizing CAP-miRSeq version 1.1 workflow [30]. CAP-miRSeq generates raw and normalized expression counts for both known mature miRNAs and predicted novel miRNAs from unaligned reads (FASTQ). Differential miRNA expression analysis was performed using bioinformatics R package edgeR to identify miRNA upregulated in DKD-MSC compared to Control-MSC (|log2FC|> 1 | and Benjamini–Hochberg adjusted *p* value < 0.05).

### Integrated mRNA/miRNA analysis

The miRNA/mRNA interactions were performed by crossing DE MSC mRNAs and miRNAs to predict targets analyzed using microRNA-Target analysis module in Ingenuity Pathway Analysis software (Ingenuity® Systems, www.ingenuity.com).

### Validation of mRNA and miRNA by quantitative polymerase chain reaction (qPCR)

To validate the expression levels of representative upregulated and down-regulated mRNAs and miRNAs, qPCR was performed on MSC. RNA was isolated from MSC (0.5–1.0 × 10^6^ cells) and ran using an Applied Biosystems ViiA7 Real-Time PCR system as previously mentioned [21]. Fold change of gene expression was calculated using 2−ΔΔCT method. All probes were from Thermo Fisher Scientific (bone morphogenetic protein 2/*BMP2*: Hs00154192, proenkephalin/*PENK*: Hs00175049, vascular cell adhesion molecule 1/*VCAM1*: Hs01003372, insulin-like growth factor-binding protein 2/*IGFBP2*: Hs01040719_m1, thrombospondin 1/*THBS1*: Hs00962908, integrin subunit beta 8/*ITGB8*: Hs00174456, glyceraldehyde 3-phosphate dehydrogenase/*GAPDH*: Hs02786624, miR-let-7a-5p: hsa-let-7a-5p, miR-30c-5p: hsa-miR-30c and *U6*: U6 small nuclear RNA/snRNA, 715680. mRNAs and miRNAs were normalized to *GAPDH* and *U6*, respectively.

### MSC angiogenic activity

Angiogenic potential of MSC was assessed in vitro. Briefly, commercially available human umbilical vein endothelial cells (HUVEC, 200K-05f, Cell Applications Inc., San Diego, CA) were grown in endothelial cell growth medium [20, 31]. HUVEC were divided into 4 groups for experimentation: group 1 cells were cultured under normal conditions (non-injured), while group 2–4 cells were co-incubated with high glucose (HG, 25 mmol) and indoxyl sulfate (IS, 1 mmol) for 6 h to simulate a uremic, diabetic milieu. Groups 3 and 4 HUVEC were simultaneously incubated with Control-MSCcm or DKD-MSCcm (medium from 1.3 × 10^5^ cells), respectively, at the time of HG + IS injury. After 6 h, all HUVEC underwent *functional* assessments in vitro (migration, proliferation, or tube formation assay) or were lysed and prepared for qPCR analyses.

HUVEC (1.6 × 10^5^/well) were seeded onto a 6-well plate and treated as described above. To assess proliferation of HUVEC in groups 1–4, the cells were fixed in 4% paraformaldehyde for 15 min, then stained for Ki67 (Abcam, ab15580) according to the manufacturers protocol. Treated HUVEC were also assayed for migration capacity via the QCM 24-well Colorimetric Cell Migration (Millipore, ECM 508) following manufacturer’s protocol. Samples were observed and imaged using an EVOS M5000 imaging system and analyzed via Image J. HUVEC (1.4 × 10^4^/well) were also seeded onto a 96-well plate coated with GelTrex™ matrix (A1413201; Thermo Fisher Scientific) to assess tube formation. Plates were placed into a 37 °C incubator with 5% CO_2_. After 4 h, HUVEC were stained with calcein-AM (10 µg/mL, for 30 min at 37 °C) to assess capillary-like structures and observed under an EVOS M5000 imaging system (Objective 40×; lens EVOS_AMEP4683; Camera 3.2 megapixels, monochrome, CMOS; Detectors and filters model DAPI—AMEP4650, F1420-1404-0373, TX Red—AMEP4655, F1620-1475-0197). A total number of tubular structures were manually counted. Gene expression was evaluated in HUVEC using the following probes from Thermo Fisher Scientific (E-selectin/*SELE*: Hs00174057, *VCAM1*, intercellular adhesion molecule 1/*ICAM-1*: Hs00164932, *VEGF*: HS00900055_m1, and *THBS1:*HS00962908_m1). Expression was normalized to TATA-binding protein (*TBP*: HS00427620).

### Statistical analysis

Data analysis was performed using GraphPad Prism 9 statistical software. Shapiro–Wilk normality test was performed on data from each group before statistical evaluation. Normally distributed data were represented as mean ± standard deviation, and non-normal data as median and interquartile range. Comparisons between Control-MSC and DKD-MSC were performed using either a two-sample t test with a 5% type-I error rate or Wilcoxon Rank Sum, as appropriate. One-way analysis of variance (ANOVA) and post hoc pairwise testing were employed to detect differences in co-culture experiments. Statistical significance was accepted at *p* ≤ 0.05.

## Results

### Baseline characteristics

In total, 38 participants were included (*n* = 9 controls and *n* = 29 DKD subjects). Clinical characteristics of each cohort are summarized in Table 1. No significant differences were evident between groups for age, sex, or race. Body mass index (BMI) was higher (35.4 ± 5.6 vs. 28.8 ± 3.6 kg/m^2^; *p* < 0.0001), and by design, eGFR was lower in DKD participants (38.9 ± 15.4 vs. 80.5 ± 13.3 mL/min/1.73 m^2^; *p* < 0.0001) compared to non-diabetic, non-CKD control subjects.

**Table 1:** Baseline characteristics in control and diabetic kidney disease subjects

	Control (*n* = 9)	DKD (*n* = 29)	*p* value
Demographics			
Age, years	64.3 ± 3.7	65.4 ± 8.0	0.59
Female sex	44.4%	31.0%	0.69
White race	100%	82.8%	0.31
Clinical			
eGFR (ml/min/1.73 m^2^)	79.0 (23.2)	36.0 (22.4)	< 0.0001
Glucose (mg/dL)	111.0 (24.0)	150.0 (82.3)	< 0.001
HbA1c (%)	–	7.7 (1.0)	–
BMI (kg/m^2^)	28.8 ± 3.6	35.4 ± 5.6	< 0.0001
Medications			
Insulin	–	72.4%	–
Oral hypoglycemics	–	55.2%	–
Insulin and oral	–	31.0%	–

Data are represented as mean ± standard deviation, median (IQR) or %

*DKD* diabetic kidney disease, *eGFR* estimated glomerular filtration rate, *HbA1c* hemoglobin A1c, *BMI* body mass index

### AD-MSC express putative surface markers

**Fig. 1:**
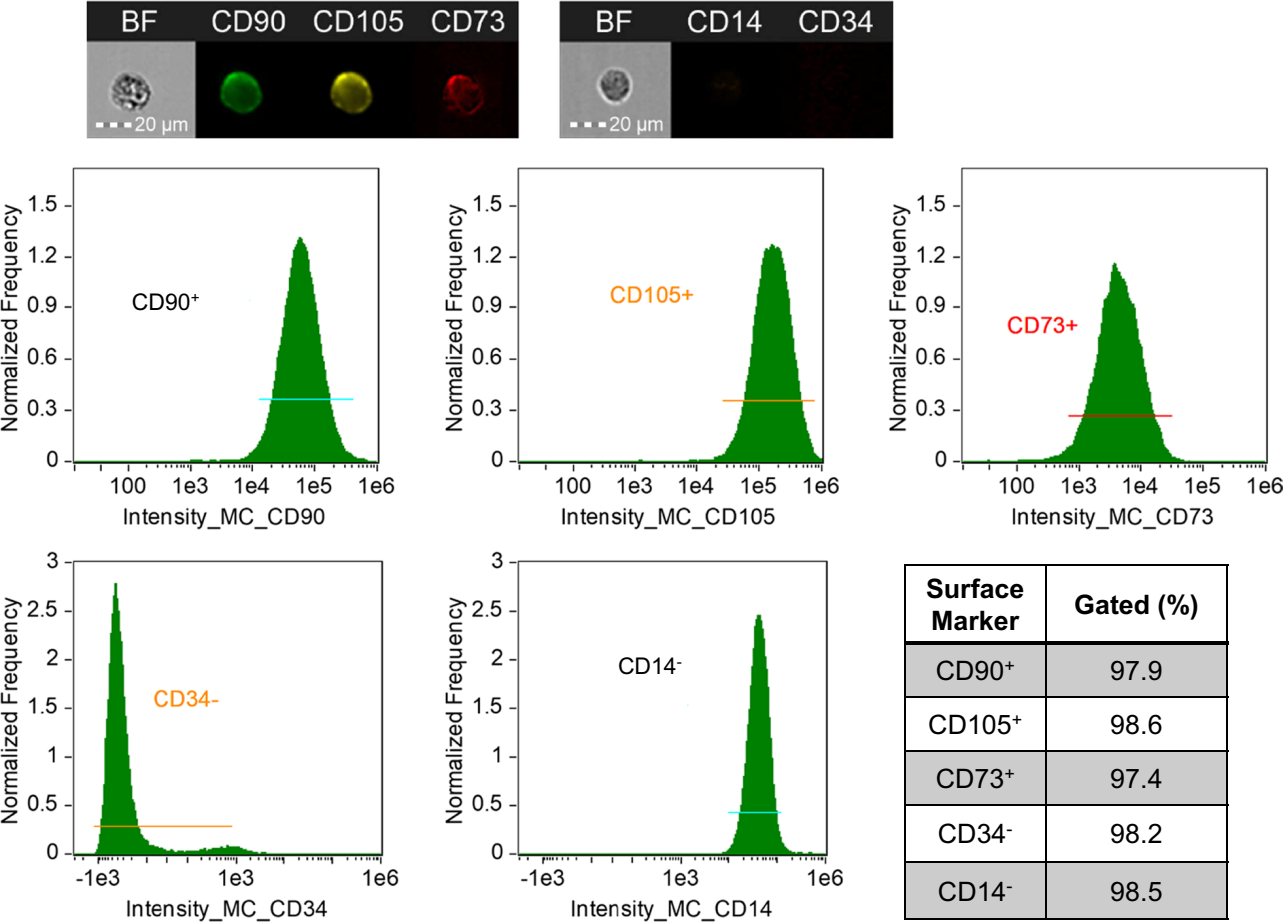
Flow cytometric characterization of passage 3 mesenchymal stromal cells (MSC) isolated from adipose tissue of diabetic kidney disease (DKD) subjects which were positive for MSC-specific surface markers cluster of differentiation (CD)90, CD105, and CD73 and were negative for hematopoietic cell markers, CD34 and CD14. BF, Brightfield

Undifferentiated AD-MSC obtained from 38 study participants produce a relatively homogenous cell population exhibiting plastic adherence, fibroblast-like morphology, and multi-lineage potential as previously shown [21]. Culture-expanded MSC demonstrated robust MSC-specific cell surface positivity to CD90, CD105, and CD73, while low expression of hematopoietic markers, CD34 and CD14, was observed in MSC from control and DKD subjects (Fig. 1). Flow cytometric analyses revealed that MSC from DKD subjects maintain surface marker characteristics meeting the criteria required for MSC [32].

### mRNA alterations in DKD-MSC

**Fig. 2:**
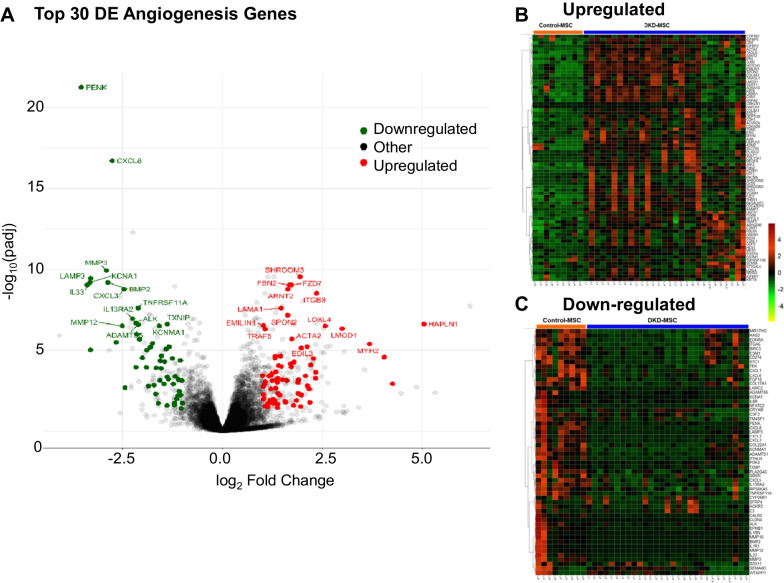
The volcano plot demonstrates the distribution of the top 30 differentially expressed (DE) upregulated (red) and down-regulated (green) angiogenesis-related genes (**A**). Heatmaps are shown for DE upregulated (**B**) and down-regulated (**C**) mRNAs in DKD-MSC. padj: adjusted *p* value

A total of 4611 mRNAs associated with angiogenesis were identified from the GeneCards® database (https://www.genecards.org/). Of these mRNAs, a total of 133 DE genes were related to angiogenesis. The distribution of DE upregulated and down-regulated genes is displayed in a volcano plot (Fig. 2A). Among them, 77 mRNAs were upregulated (Fig. 2B) and 56 down-regulated in DKD-MSC (Fig. 2C). Among the top 15 DE upregulated mRNAs were pro-angiogenic genes, including *IGFBP2*, lysyl oxidase-like 4 (*LOXL4*) and *VCAM1*, and anti-angiogenic genes, including *THBS1* and *ITGB8*. Among the top 15 DE down-regulated genes several were pro-angiogenic genes, including interleukin (*IL*)13 receptor subunit alpha-2, C-X-C motif chemokine ligand (*CXCL*)*3*, *IL33*, *PENK*, matrix metalloproteinase (*MMP*)*10*, *CXCL8*, *BMP2*, and *MMP3* as well as an anti-angiogenic gene, *MMP12*. Expression patterns of several mRNAs were subsequently confirmed by qPCR (Additional file 1: Fig. S1A and C). In addition, DKD-MSCcm tended to release higher levels of the anti-angiogenic protein TSP1 compared to Control-MSCcm (Additional file 1: Fig. S1B).

### miRNA alterations in DKD-MSC

**Fig. 3:**
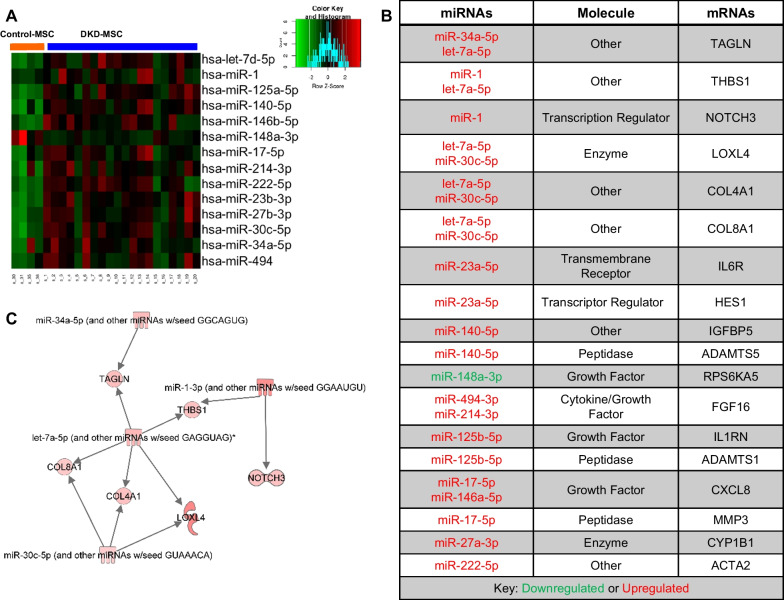
Heatmap demonstrates 14 DE microRNAs (miRNAs) in DKD-MSC (**A**). Table lists differentially expressed miRNA and their potential angiogenesis-related mRNA targets analyzed by ingenuity pathway analysis (**B**). Font colors: upregulated (red), down-regulated (green) miRNAs. Diagram of let-7a-5p targeting of several mRNAs including TAGLN, THBS1, LOXL4, COL4A1, and COL8A1 in addition to other differentially expressed miRNA activities (**C**)

A total of 575 annotated miRNAs were identified, of which 208 (119 upregulated and 89 down-regulated) were DE in DKD-MSC. Expression patterns of miRNAs were subsequently confirmed by qPCR (Additional file 1: Fig. S1D). DE miRNAs were further inspected to identify mRNA targets involved in angiogenesis. Target prediction analysis resulted in 14 unique DE miRNAs regulating 18 unique DE angiogenesis-related genes (Fig. 3A) and 25 mRNA-miRNA interactions (Fig. 3B). Surprisingly, only one miRNA was down-regulated (miR-148a-3p), which interacted with ribosomal protein S6 kinase A5 (*RPS6KA5*), involved in phosphorylating cAMP-response element-binding protein (*CREB*) that promotes angiogenesis by regulating vascular endothelial growth factor (VEGF) receptor-1(*VEGFR1*). Notably, let-7a-5p targeted five mRNAs (transgelin/*TAGLN*, *THBS1*, *LOXL4*, collagen 4A1/*COL4A1*, and collagen 8A1/*COL8A1*; Fig. 3C).

### DKD-MSCcm angiogenic functionality

**Fig. 4:**
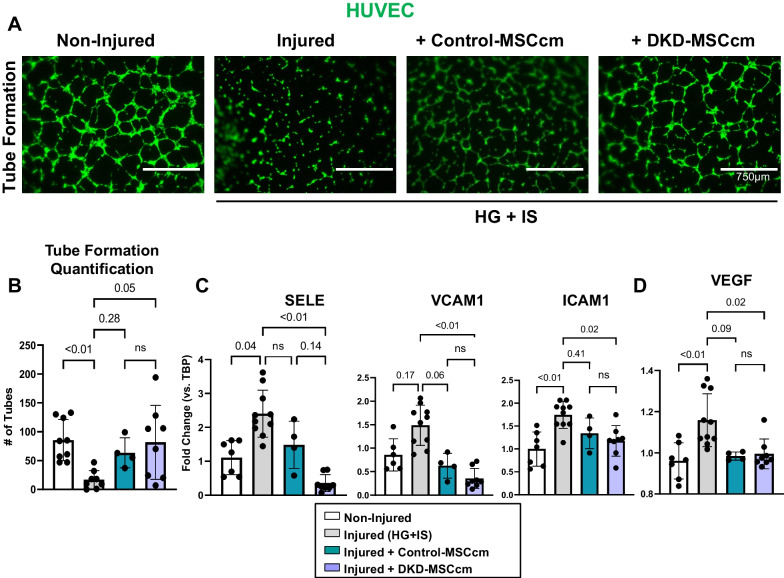
Representative images of capillary-like tubes formed by non-injured human umbilical vein endothelial cells (HUVEC) or in the presence of high glucose (HG) plus indoxyl sulfate (IS) injury and either Control-MSC conditioned medium (cm) or DKD-MSCcm. Images were acquired at 40× resolution and were not enhanced. (**A**). Quantification of total number of tubes among groups (**B**). Selectin E (*SELE*), vascular cell adhesion molecule 1 (*VCAM1*), intercellular adhesion molecule 1 (*ICAM-1)* (**C**) and vascular endothelial growth factor (*VEGF*) (**D**) mRNA expression levels in HUVEC. ns, not statistically significant (*p* > 0.5); other non-significant *p* values (*p* > 0.05) are shown in this figure given trends in the relationships

Apart from the influence of hyperglycemia, uremic conditions present in DKD may further impact gene expression, intrinsic properties of MSC, and MSC functionality. Therefore, we assessed the reparative capacity of DKD-MSCcm on injured endothelial cells by establishing a uremic and diabetic microenvironment using HG + IS. Pre-incubation of HUVEC with HG + IS diminished tube formation capacity. Co-incubation of HUVEC with DKD-MSCcm prior to HG + IS exposure restored formation of tube-like networks (Fig. 4A, B), reflecting sustained pro-angiogenic properties. In other functional studies, incubation of DKD-MSCcm restored the migration potential of injured HUVEC, while proliferation activity was not different between groups (Additional file 2: Fig. S2A, B). Co-culture of MSCcm with injured HUVEC increased THBS1 mRNA expression, though DKD-MSCcm exhibited lower THBS1 levels when compared with Control-MSCcm (*p* = 0.07) (Additional file 2: Fig. S2C). Furthermore, exposure to HG + IS led to upregulation of adhesion molecules *SELE*, *VCAM1*, *ICAM-1* in HUVEC, while incubation of DKD-MSCcm attenuated mRNA expression, suggesting that DKD-MSCcm prevents endothelial dysfunction (Fig. 4C). No differences were observed between DKD-MSCcm and Control-MSCcm groups. Furthermore, VEGF mRNA levels increased in HG + IS-exposed HUVEC, but MSCcm from DKD and Controls prevented the rise in VEGF expression (Fig. 4D).

## Discussion

Our study used comprehensive sequencing analysis to compare the enrichment and suppression of mRNA and miRNA expression in adipose tissue-derived MSC in DKD and control subjects. We further explored the pro-angiogenic potential of DKD-MSCcm in vitro. Compared to MSC from controls, DKD-MSC exhibited heterogeneity in the expression profiles of mRNA associated with angiogenic activity. Furthermore, 14 miRNAs targeted these angiogenesis-related genes modifying mRNA activity. In particular, miRNA let-7a-5p, which regulates angiogenesis, has anti-thrombospondin activity, and is associated with DKD pathogenesis, was upregulated and had the highest number of mRNA targets. In our in vitro studies mimicking the DKD microenvironment, DKD-MSCcm incubation restored endothelial cell function and halted overexpression of adhesion molecules associated with vascular insult. These observations suggest that despite alterations of the transcriptome, the pro-angiogenic reparative capacity of MSC in vitro appears relatively preserved in DKD subjects.

To limit potential immunological reactions and minimize the risk of allosensitization in subjects who may eventually desire kidney transplantation, an autologous MSC source is preferable. However, DKD patient factors such as advanced age, hyperglycemia, and uremia may diminish MSC reparative ability. Our previous ex vivo studies in MSC from patients with DKD showed that DKD-MSC anti-inflammatory, immunomodulatory, anti-apoptosis, and anti-fibrosis functionality remained intact compared with Control-MSC. However, the migratory capacity of DKD-MSC was lower than that of Control-MSC [21]. Therefore, we further explored functional differences in angiogenic capacity between DKD-MSC and Control-MSC in this study.

Angiogenic activity is dependent on the intricate balance between pro-angiogenic factors (such as VEGF, fibroblast growth factor-2/FGF2, transforming growth factor-β/TGF-β, and angiopoietins) and factors that inhibit angiogenesis (angiostatin, endostatin, thrombospondins) [33]. Diabetes is associated with angiogenesis dysregulation leading to structurally immature blood vessel formation after injury, impaired wound healing, and excessive angiogenesis—particularly in DKD and diabetic retinopathy [33, 34]. We previously found no difference in secretion of VEGF-A between DKD-MSC and Control-MSC, while hepatocyte growth factor (HGF), maintaining anti-apoptosis, anti-fibrotic, and pro-angiogenic activity, was substantially higher in DKD-MSC [21]. VEGF is the key mediator of angiogenesis and vessel repair. Xin et al. [35] found that the combination of HGF and VEGF increased neovascularization greater than either growth factor alone. In our studies, DKD-MSCcm restored HUVEC formation of tube-like networks and migration ability in a uremic, diabetic milieu, thus demonstrating that DKD-MSC maintain angiogenic repair capability in vitro. Previous studies identified MSC subsets possessing greater angiogenic paracrine activity. Du et al. [36] found that VCAM1^+^ expressing MSC had increased gene expression and release of pro-angiogenic factors and cytokines (VEGF, HGF, MMP2, and CXCL1) compared to VCAM1^−^-MSC. In in vivo studies, exogenous delivery of VCAM1^+^-MSC increased perfusion in the preclinical ischemic hindlimb model versus VCAM1^−^-MSC. Likewise, VCAM1 mRNA was upregulated in our DKD-MSC, which could potentially influence pro-angiogenic repair activity observed in vitro.

Contrarily, anti-angiogenic genes were upregulated (e.g. THBS1) and pro-angiogenic genes were down-regulated (e.g. BMP2) in DKD-MSC in our study, which could disturb angiogenic activity. Thrombospondin 1 (TSP1), encoded by the *THBS1* gene, is a secreted anti-angiogenic protein that modulates cell migration and adhesion by regulating vascular nitric oxide signaling [37, 38]. In a study by Dzhoyashvili et al. [39], MSC harvested from subjects with type 2 diabetes and coronary artery disease showed impaired ability to stimulate angiogenesis in vitro with significant increase in mRNA level of THBS1, as well as closely negative correlation between its mRNA level and MSC angiogenic activity measured by tube formation assay. Similar to these findings, our MSC harvested from participants with DKD also had trends of higher THBS1 mRNA expression and protein release in condition media, but no statistical differences compared with Control-MSC for angiogenic capability to restore formation of tube-like networks in injured endothelial cells. Similarly, we found that DKD-MSCcm led to increased migration of HG + IS injured HUVEC versus injured controls with no difference observed between DKD- and Control-MSCcm groups. BMP2 mRNA expression was lower in DKD-MSC in our study. BMP2 has been proven to promote angiogenesis by facilitating endothelial migration [40], invasion, and proliferation [41] and induce HUVEC tube formation [42]. Overall, these observations suggest that the ultimate functional outcome may depend on a weighted effect of these genes and their inhibitors, in concert with the impact of the microenvironment.

Given that miRNA regulate gene activity, we further assessed the relationship between miRNA and angiogenesis-related mRNA in DKD-MSC. In our study, 208 differentially expressed miRNAs were identified among which 14 miRNAs targeted differentially expressed angiogenesis-related mRNA. Interestingly, let-7a-5p was upregulated in RNA-seq analysis (and had a trend toward higher gene expression, supplemental Fig. 1D) and targets the highest number of mRNAs, including *TAGLN*, *THBS1*, *LOXL4*, *COL4A1*, and *COL8A1*. Let-7a-5p has pro-angiogenic properties with increasing sprout formation of hypoxic HUVEC [43, 44] by directly targeting the 3′UTR of the THBS1 mRNA [45]. Hence, we speculate that DKD-MSC restore angiogenetic abilities partly because of increased let-7a-5p. Zhu et al. [44] conducted miRNA sequencing revealing that let-7 family (let-7a-5p) was enriched in EVs derived from human AD-MSC, and blocking of let-7 impaired tube formation of HUVEC in vitro. In addition, platelet-derived EVs can deliver let-7a into HUVEC in vitro with inhibition of HUVEC tube formation by down-regulating TSP1 protein expression [45]. Consistent with these findings, we used DKD-MSCcm, containing MSC-EVs and found trends of higher tube formation and lower THBS1 mRNA expression in HUVEC compared to Control-MSCcm. Further investigation is required to explore the mechanism and outcomes of altered let-7a-5p in vivo and in vitro. In addition, our studies found that TSP1 targets included: *TAGLN*, a regulator of angiogenesis [46], *LOXL4*, a contributor to the vascular permeability in diabetes [47], types of collagen (*COL4A1* and *COL8A1)* that influence angiogenesis plus *THSB1* an inhibitor of angiogenesis [48]. Further observations are needed to explore how let-7a-5p adjusts these mRNA. Taken together, these observations suggest that differentially expressed miRNAs, particularly let-7a-5p, in DKD-MSC might contribute to angiogenesis dysregulation.

We further examined MSC angiogenic repair capacity in vitro. After an in vitro injury mimicking a DKD microenvironment, endothelial cells lost tube formation capacity and increased adhesion molecule (*SELE, VCAM, ICAM-1)* expression, key molecules associated in early vascular injury [49–51]. These effects were reversed following co-incubation with DKD-MSCcm. Additionally, HUVEC migration was reduced after HG + IS treatment but improved with DKD-MSCcm co-culture but was not different versus Control-MSCcm. Angiogenic function impairment in vivo was shown by Kim et al*.* [52] wherein exogenous delivery of MSC from diabetic rats failed to improve blood flow compared to MSC from non-hyperglycemic rats in a model of hindlimb ischemia. Interestingly, higher expression levels of pro-angiogenic factors (HGF, FGF2, PGF, insulin-like growth factor-1*,* and angiopoietin-2 were evident in both control- and diabetic MSC infused into ischemic tissue 2 weeks-post administration, indicating that diabetic MSC still possess paracrine angiogenic repair capacities albeit limited. Hence, a therapeutic boost of autologous MSC infusion could still help enhance angiogenic repair capacity in patients with DKD.

This study has limitations. First, the predominantly Caucasian and relatively older study cohort may limit generalizability of our findings to other races and younger individuals with DKD, but remains reflective of the DKD population in the USA [53]. Secondly, AD-MSC may differ from other autologous cell sources, such as bone marrow or umbilical cord [54], though AD-MSC may possess more potent pro-angiogenic activity [26]. Furthermore, in vivo studies are needed to confirm these in vitro findings in DKD-MSC. Finally, future mechanistic studies employing loss of function (e.g., siRNA or miRNA inhibitors) are necessary to establish which DE genes are involved in modification of DKD-MSC angiogenic function.

## Conclusions

Compared to Control-MSC, AD-MSC harvested from DKD participants possess modified genetic messages related to angiogenesis. Yet, DKD-MSC maintained the intrinsic capacity to release pro-angiogenic paracrine factors to avert further endothelial dysfunction. Identification of disease-related dysfunction and interindividual variation in MSC may allow for development of regenerative strategies to improve MSC function and thwart barriers that prevent successful MSC therapeutic outcomes in individuals with DKD.

## Supplementary Information

**Additional file 1: Fig. S1.** Validation of upregulated (**A**) and down-regulated (**C**) differentially expressed (DE) messenger RNAs (mRNAs) in diabetic kidney disease (DKD)-mesenchymal stromal cells (MSC), as well as microRNAs (miRNAs) (**D**). ELISA of TSP1 in MSC conditioned medium (MSCcm) (**B**). *BMP2*, bone morphogenetic protein 2; *PENK*, proenkephalin; *VCAM1*, vascular cell adhesion molecule 1; *IGFBP2*, insulin-like growth factor-binding protein 2; *THBS1/TSP1*, thrombospondin 1; *ITGB8*, integrin subunit beta 8.

**Additional file 2: Fig. S2.** Representative images of capillary-like tubes formed by non-injured human umbilical vein endothelial cells (HUVEC) or in the presence of high glucose (HG) plus indoxyl sulfate (IS) injury and either Control-MSC conditioned medium (cm) or DKD-MSCcm. Images were acquired at 40X resolution and were not enhanced. (**A**). 4′,6-diamidino-2-phenylindole (DAPI) nuclear DNA (**A**). Nuclear staining (blue), proliferation marker Ki67 protein staining (red). Quantification of proliferation ability (**A**) and migratory function (**B**). Thrombospondin (*THSB1*) gene expression in HUVEC groups (**C**).

## Data Availability

The datasets supporting the conclusions of this article are available from the corresponding author on reasonable request, without undue reservation. Sequencing data were deposited in NCBI’s Gene Expression Omnibus and are publicly available [accession number: GSE217712].

## Abbreviations

AD-MSC: Adipose tissue-derived mesenchymal stem cells
ANOVA: Analysis of variance
BMP2: Bone morphogenetic protein 2
BMI: Body mass index
CD: Cluster of differentiation
CKD: Chronic kidney disease
CM: Conditioned medium
COL4A1: Collagen 4A1
COL8A1: Collagen 8A1
Control-MSC: Control mesenchymal stromal cells
CREB: CAMP-response element-binding protein
CXCL: C–X–C motif chemokine ligand
DE: Differentially expressed
DKD: Diabetic kidney disease
DKD-MSC: Diabetic kidney disease mesenchymal stromal cells
DKD-MSCcm: Diabetic kidney disease mesenchymal stromal cells condition medium
eGFR: Estimated glomerular filtration rate
FC: Fold change
FGF2: Fibroblast growth factor-2
GAPDH: Glyceraldehyde 3-phosphate dehydrogenase
HbA1c: Hemoglobin A1c
HG: High glucose
HGF: Hepatocyte growth factor
HUVEC: Human umbilical vein endothelial cells
ICAM-1: Intercellular adhesion molecule 1
IGFBP2: Insulin-like growth factor-binding protein 2
IL13RA2: Interleukin 13 receptor subunit alpha-2
IQR: Interquartile range
IS: Indoxyl sulfate
ITGB8: Integrin subunit beta 8
LOLX4: Lysyl oxidase-like 4
MAP-RSeq: Mayo analysis pipeline for RNA-seq
miRNA: MicroRNA
MMP: Matrix metalloproteinase
mRNA: Messenger RNA
MSC: Mesenchymal stromal cells
MSCcm: Mesenchymal stromal cells condition medium
padj: Adjusted *p* value
PAI-1: Plasminogen activator inhibitor-1
PENK: Proenkephalin
PGF: Placental growth factor
RNA-seq: RNA sequencing
RPS6KA5: Ribosomal protein S6 kinase A5
SELE: Selectin E
snRNA: Small nuclear RNA
TAGLN: Transgelin
TBP: TATA-binding protein
TGF-β: Transforming growth factor-β
THBS1: Thrombospondin 1 (gene)
TSP1: Thrombospondin 1 (protein)
VCAM1: Vascular cell adhesion molecule 1
VEGF: Vascular endothelial growth factor
VEGFR1: Vascular endothelial growth factor receptor-1
