# Supplementary material for: Diabetic kidney disease induces transcriptome alterations associated with angiogenesis activity in human mesenchymal stromal cells
Source: Stem Cell Res Ther. 2023 Mar 22;14:49. doi: 10.1186/s13287-023-03269-9 (PMC10035152; doi:10.1186/s13287-023-03269-9)
Supplement: Supplementary file 1 — Additional file 1: Fig. S1. Validation of upregulated (A) and down-regulated (C) differentially expressed (DE) messenger RNAs (mRNAs) in diabetic kidney disease (DKD)-mesenchymal stromal cells (MSC), as well as microRNAs (miRNAs) (D). ELISA of TSP1 in MSC conditioned medium (MSCcm) (B). BMP2, bone morphogenetic protein 2; PENK, proenkephalin; VCAM1, vascular cell adhesion molecule 1; IGFBP2, insulin-like growth factor-binding protein 2; THBS1/TSP1, thrombospondin 1; ITGB8, integrin subunit beta 8. [file 13287_2023_3269_MOESM1_ESM.pptx]

## Slide 1
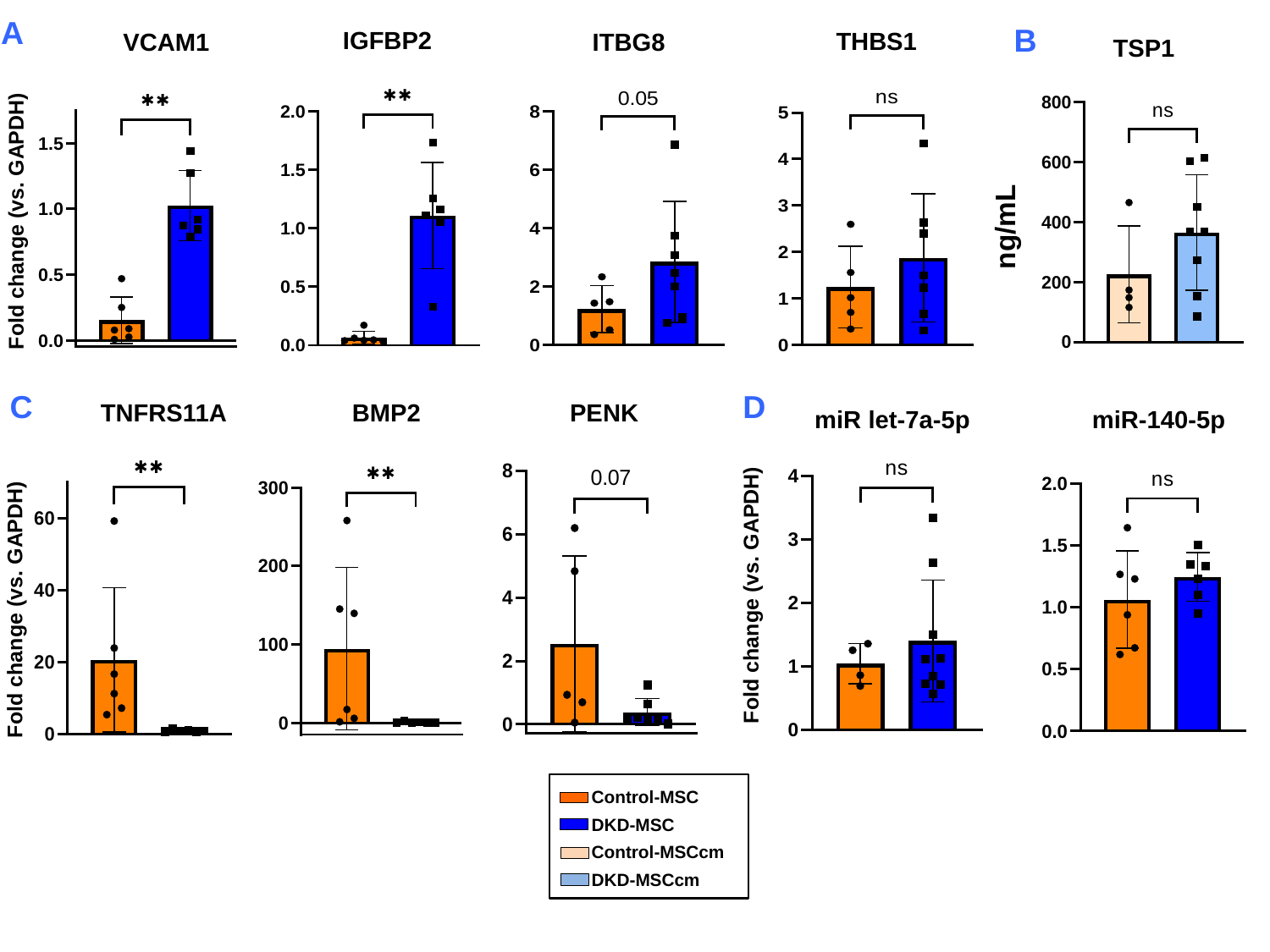

A
B
IGFBP2
THBS1
VCAM1
ITBG8
TSP1
Fold change (vs. GAPDH)
ng/mL
C
D
TNFRS11A
BMP2
PENK
miR let-7a-5p
miR-140-5p
Fold change (vs. GAPDH)
Fold change (vs. GAPDH)
Control-MSC
DKD-MSC
Control-MSCcm
DKD-MSCcm
