# Supplementary material for: Diabetic kidney disease induces transcriptome alterations associated with angiogenesis activity in human mesenchymal stromal cells
Source: Stem Cell Res Ther. 2023 Mar 22;14:49. doi: 10.1186/s13287-023-03269-9 (PMC10035152; doi:10.1186/s13287-023-03269-9)
Supplement: Supplementary file 2 — Additional file 2: Fig. S2. Representative images of capillary-like tubes formed by non-injured human umbilical vein endothelial cells (HUVEC) or in the presence of high glucose (HG) plus indoxyl sulfate (IS) injury and either Control-MSC conditioned medium (cm) or DKD-MSCcm. Images were acquired at 40X resolution and were not enhanced. (A). 4′,6-diamidino-2-phenylindole (DAPI) nuclear DNA (A). Nuclear staining (blue), proliferation marker Ki67 protein staining (red). Quantification of proliferation ability (A) and migratory function (B). Thrombospondin (THSB1) gene expression in HUVEC groups (C). [file 13287_2023_3269_MOESM2_ESM.pptx]

## Slide 1
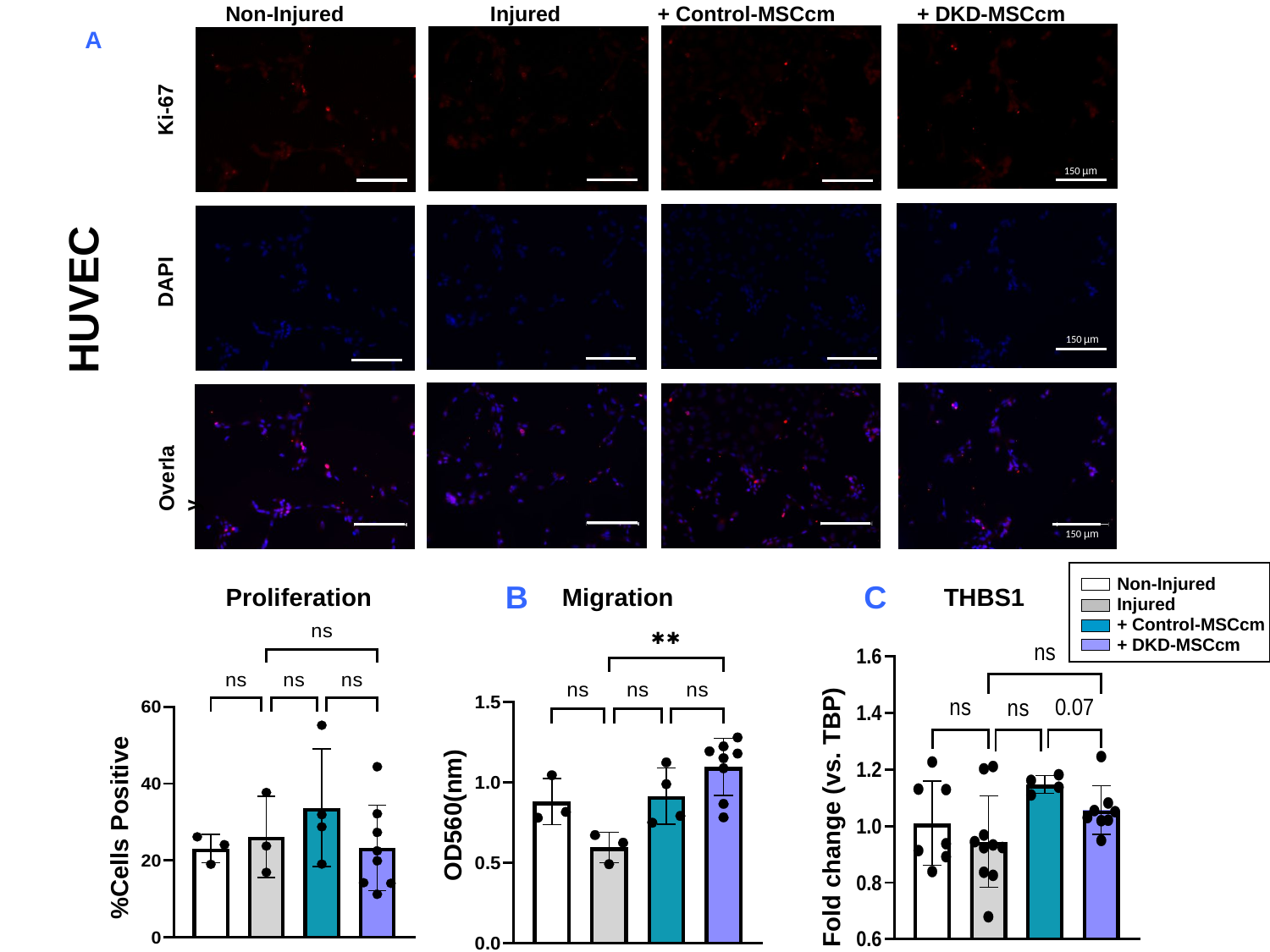

Non-Injured
Injured
+ Control-MSCcm
+ DKD-MSCcm
A
Ki-67
A
B
D
C
150 µm
HUVEC
DAPI
150 µm
150 µm
Overlay
Non-Injured
Injured
+ Control-MSCcm
+ DKD-MSCcm
B
C
Proliferation
Migration
THBS1
Fold change (vs. TBP)
OD560(nm)
%Cells Positive
